# Basilar invagination in osteogenesis imperfecta—Case report

**DOI:** 10.1016/j.radcr.2025.06.048

**Published:** 2025-07-12

**Authors:** Aviyani Aviyani, Raisa Mahmudah

**Affiliations:** Department of Radiology, Faculty of Medicine, Universitas Padjadjaran, West Java, Indonesia

**Keywords:** Osteogenesis imperfecta, Basilar invagination

## Abstract

Osteogenesis imperfecta (OI) is a genetic disorder characterized by defective type I collagen synthesis, leading to fragile bones and skeletal deformities. One of the serious complications of OI involves abnormalities of the craniovertebral junction, including basilar invagination, basilar impression, and platybasia, which can cause skull base deformities and brainstem compression. We report a case of a 19-year-old patient with OI presenting with headaches, neurological deficits, and visual impairment, whose contrast-enhanced head CT scan revealed basilar invagination, fusion of midbrain colliculi, tonsillar herniation into the foramen magnum, and communicating hydrocephalus. These cranial abnormalities arise from weakened bone structures inherent in OI and may disrupt cerebrospinal fluid flow, leading to significant neurological symptoms. Early detection and ongoing monitoring of craniovertebral anomalies in OI patients are essential to prevent severe complications and improve clinical outcomes.

## Introduction

Osteogenesis imperfecta (OI) is a hereditary connective tissue disorder resulting from defects in the production or processing of type I collagen. The condition leads to fragile bones that fracture easily and reduced bone density [1]. Alterations in bone structure associated with OI can also involve the craniocervical or craniovertebral junction (CCJ), representing one of the most serious complications of the disorder. Basilar anomalies, present in up to 37% of cases, may lead to skull base deformities that exert pressure on the brainstem. This compression can result in significant neurological impairment and, in severe instances, may be life-threatening. Three primary anomalies are frequently observed: basilar invagination, where the odontoid process extends into the foramen magnum; basilar impression, characterized by the elevation of the odontoid process above the lower margins of the skull without entering the foramen magnum; and platybasia, defined as an abnormal flattening of the skull base [2].

This case will discuss the contrast-enhanced head CT scan findings in a 19-year-old patient with OI.

## Case report

### Case presentation

**Fig. 1:**
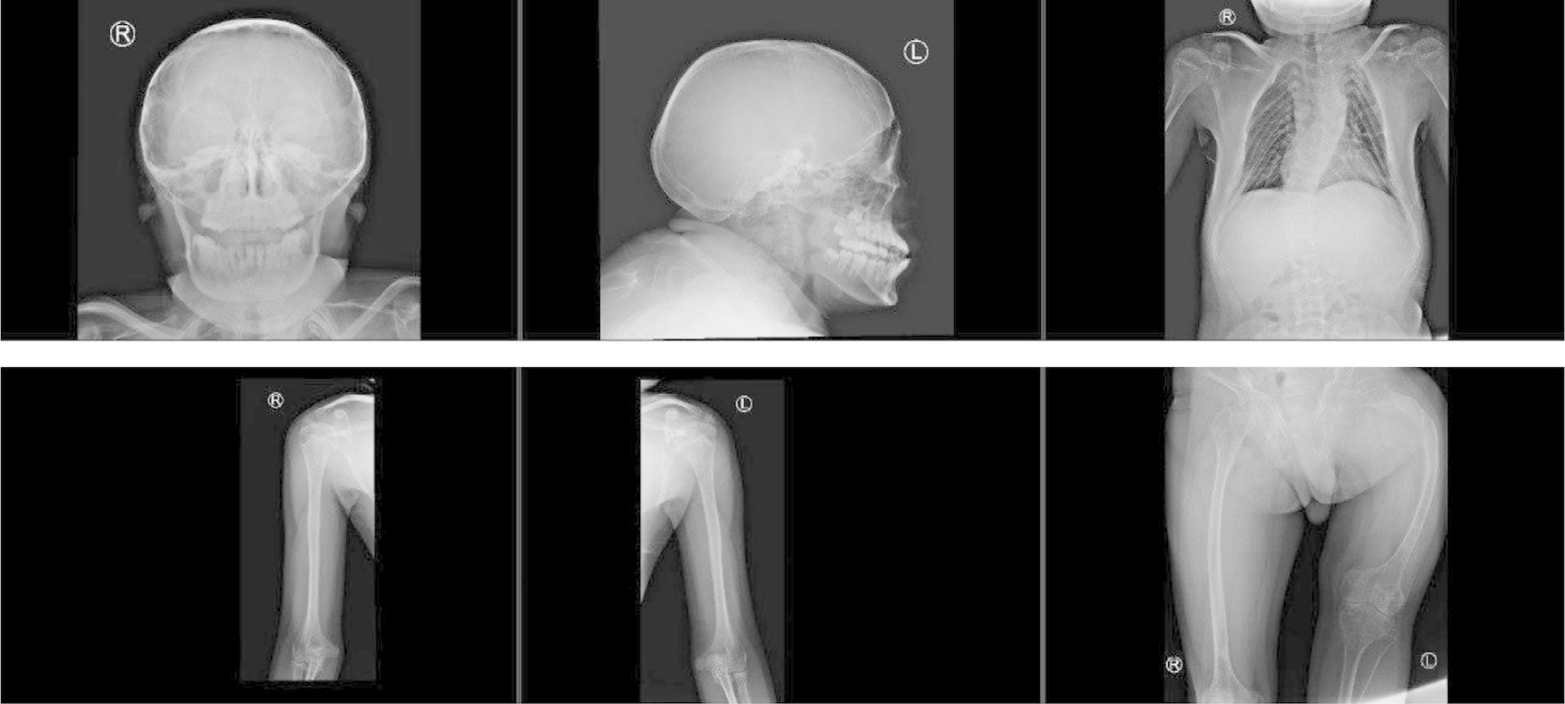
X-ray of the patient shows decreased bone density, deformities of the thoracolumbosacral vertebrae with a codfish appearance and platyspondyly, malunion of a fracture in the proximal third of the left femur, and bilateral femoral bowing, more pronounced on the left.

The patient is a 19-year-old who initially presented with a history of leg fracture 5 years ago due to a fall (Fig. 1). Since then, the patient has been bedridden. Over the past 6 months, the patient has experienced intermittent headaches described as heavy and constricting, accompanied by slurred speech and facial asymmetry. There is also a history of visual impairment in the right eye due to previous trauma (struck by the edge of a bottle cap). The diagnosis of OI in this patient was based on clinical evaluation and radiologic imaging.

## Diagnostic evaluation

**Fig. 2:**
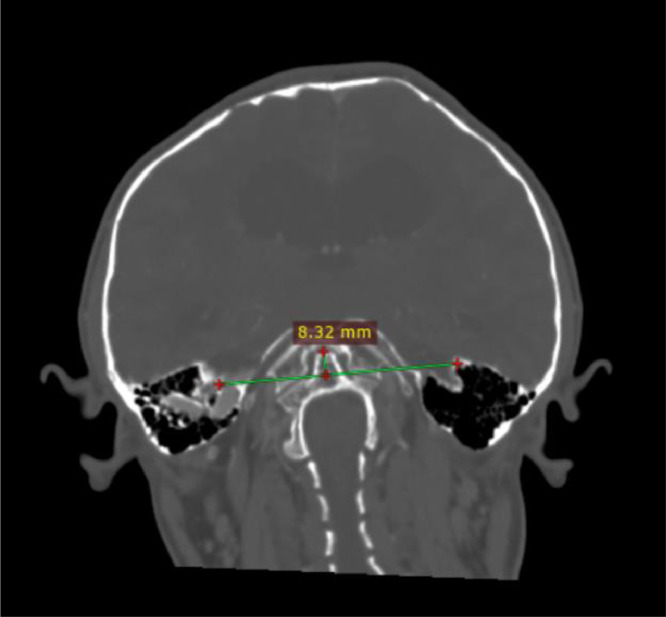
Basilar Invagination.

**Fig. 3:**
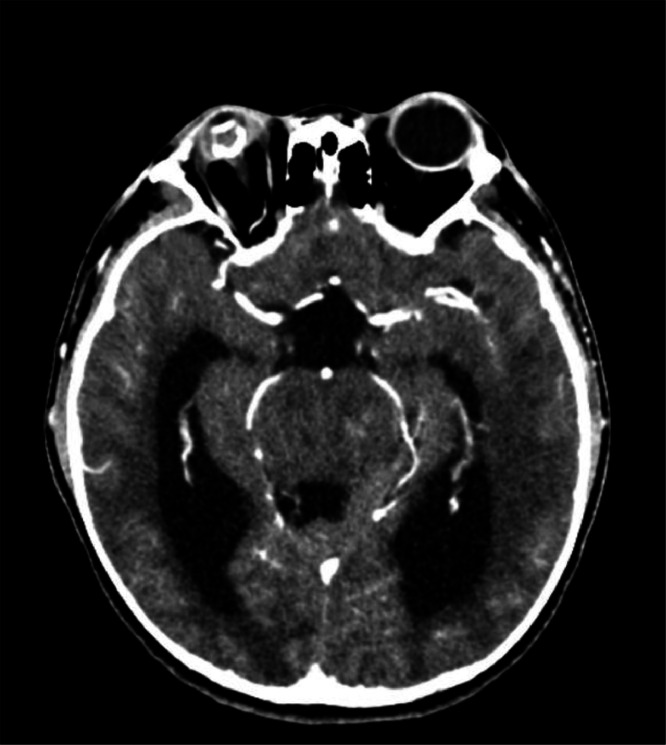
Fusion of midbrain colliculi.

**Fig. 4:**
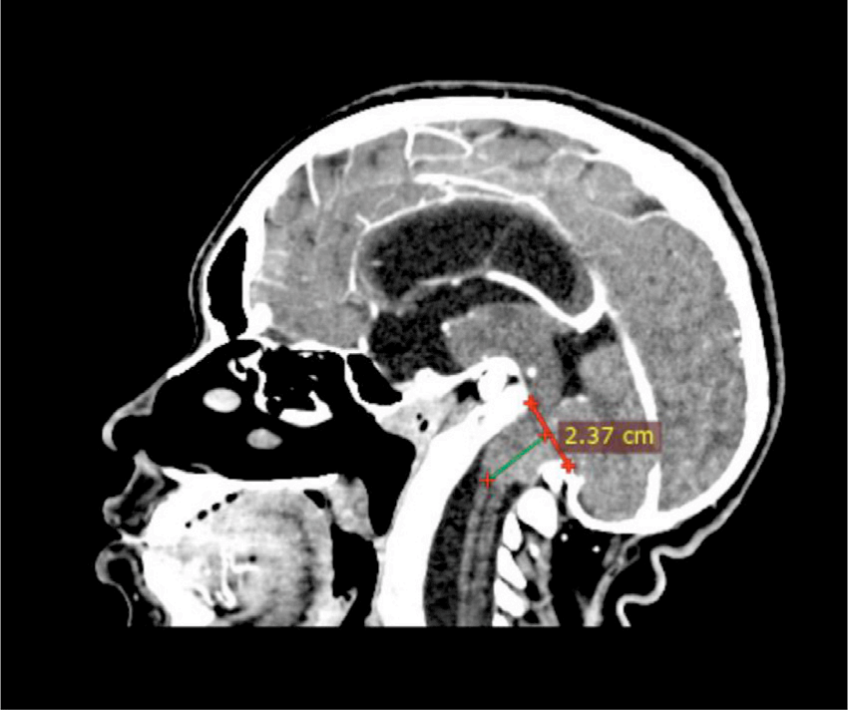
Displacement of cerebellar tonsils and vermis into the foramen magnum.

**Fig. 5:**
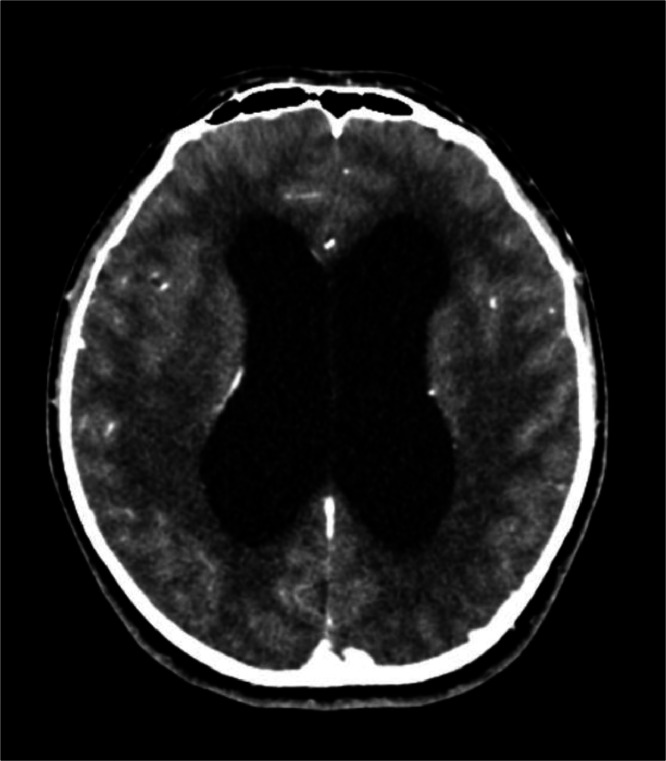
Hydrocephalus.

The patient underwent a contrast-enhanced head CT scan for the first time. The findings revealed that the tip of the odontoid process was located 8.32 mm above the digastric line (Fig. 2). There was fusion of the midbrain colliculi with posterior protrusion and invagination into the cerebellum (Fig. 3, Fig. 4). The cerebellar tonsils and vermis were displaced downward into the foramen magnum by approximately 2.3 cm. The lateral ventricles appeared asymmetric with blunting, and there was dilation of the bilateral lateral ventricles, third ventricle, and fourth ventricle (Fig. 5). The right eyeball appeared atrophic with internal calcification due to trauma (Fig. 3). The overall impression was suggestive of basilar invagination into the cerebellum accompanied by tonsillar herniation through the foramen magnum, resulting in communicating hydrocephalus. The odontoid process was positioned 0.8 cm above the digastric line. Right phthisis bulbi was also noted.

## Discussion

Basilar invagination is a significant abnormality of the craniovertebral junction commonly seen in individuals with OI, especially in those with more severe disease manifestations. In the research conducted by Arponen and colleagues, basilar invagination was identified in 13% of the subjects. The study also emphasized that craniovertebral abnormalities were found in every type of OI examined [3].

In individuals with OI, basilar invagination commonly arises due to the weakened and malformed bone structures inherent to the condition. OI involves genetic mutations that disrupt type I collagen synthesis, which undermines bone density and architectural stability. As a result, the craniovertebral junction becomes susceptible to abnormalities, such as platybasia—a flattening of the skull base—that can allow the odontoid process to shift upward into the foramen magnum, leading to basilar invagination [4].

Basilar invagination can disrupt the typical anatomy of the posterior fossa, resulting in the downward displacement of the cerebellar tonsils through the foramen magnum, a condition known as tonsillar herniation. In patients with OI, this herniation may arise from factors such as a reduced volume of the posterior fossa, upward pressure on the brainstem and cerebellum caused by basilar invagination, or the sagging of intracranial structures due to compromised bone and connective tissue support. This downward displacement can obstruct cerebrospinal fluid (CSF) flow, potentially leading to hydrocephalus. Clinically, patients may experience symptoms like headaches, nausea, vomiting, difficulties with walking, or changes in cognitive function [5,6].

OI also can be associated with significant midbrain and cerebellar anomalies. Structural defects also included cerebellar vermis hypoplasia, and in one case, fused midbrain colliculi [7].

## Conclusion

OI is associated with significant abnormalities at the craniovertebral junction, such as basilar invagination, which arises from compromised bone integrity caused by defects in type I collagen. This condition can result in the downward herniation of the cerebellar tonsils and disruption of cerebrospinal fluid circulation, potentially leading to hydrocephalus. These structural changes may profoundly affect neurological function and patient well-being, underscoring the need for prompt diagnosis and careful surveillance, particularly in individuals exhibiting neurological signs.

## Patient consent

Informed consent for publication of their case was obtained from the patient.
